# Plasma IL-6 as a shared inflammatory marker of cognitive impairment across racially and ethnically diverse older adults: matched analyses from the HABS-HD study

**DOI:** 10.3389/frdem.2026.1930196

**Published:** 2026-10-14

**Authors:** Maria B. Misiura, Tammy O. Joseph, Jessica A. Cooper, Gabi Champion, Jennalyn Burnette, Alex Bartlett, Kyle Thatcher, Nadine Ciera Austin, Vonetta M. Dotson

**Affiliations:** 1 Tri-institutional Center for Translational Research in Neuroimaging and Data Science (TReNDS), Georgia State University, Georgia Institute of Technology, and Emory University, Atlanta, GA, United States; 2 Institute for Biomedical Sciences, Georgia State University, Atlanta, GA, United States; 3 Department of Psychiatry and Behavioral Sciences, Emory University School of Medicine, Atlanta, GA, United States; 4 Department of Psychology, Georgia State University, Atlanta, GA, United States; 5 Department of Neurology, Brigham and Women’s Hospital, Boston, MA, United States; 6 Department of Neurology, Harvard Medical School, Boston, MA, United States; 7 Gerontology Institute, Georgia State University, Atlanta, GA, United States

**Keywords:** cognitive impairment (CI), cytokine—immunological terms, health disparities African-American, IL-6 (interleukin 6), inflammation

## Abstract

**Background:**

Systemic inflammation is implicated in cognitive aging, yet whether interleukin-6 (IL-6) represents a shared versus group-specific marker of cognitive impairment across racially and ethnically diverse older adults remains unclear. This study examined associations among depressive symptoms, plasma IL-6, and cognitive impairment while testing mediation and ethnoracial moderation.

**Methods:**

Participants were drawn from the Health and Aging Brain Study–Health Disparities (HABS-HD). In the full multi-ethnic complete-case sample (*N* = 2,187; 963 non-Hispanic White, 277 Black, and 947 Hispanic participants), logistic regression models tested the association of log₁₀-transformed plasma IL-6 with binary cognitive impairment (mild cognitive impairment or dementia versus cognitively unimpaired status), adjusting for age, education, sex, Geriatric Depression Scale (GDS) total score, ethnoracial group, and APOE ε4 carrier status. Parallel models examined GDS–cognitive impairment associations. Mediation models tested whether IL-6 statistically mediated the association between GDS scores and cognitive impairment. Moderation analyses tested IL-6 × ethnoracial group interactions. Sensitivity analyses repeated the main-effect, interaction, and mediation models in 1:1 matched Black/White (*N* = 554) and Hispanic/White (*N* = 794) subsamples that were matched exactly on sex and cognitive status and balanced on age and education.

**Results:**

Higher plasma IL-6 was associated with greater odds of cognitive impairment in the full sample (OR = 1.87, 95% CI [1.31, 2.68], *p* < 0.001), and this association did not significantly differ by ethnoracial group (χ^2^ = 3.52, df = 2, *p* = 0.172). Higher GDS scores were also independently associated with cognitive impairment (OR = 1.06 per point, 95% CI [1.04, 1.08], *p* < 0.001). IL-6 statistically mediated a small proportion of the GDS–cognitive impairment association (indirect effect = 0.0025, Sobel *p* = 0.014). The IL-6 association remained significant in the Black/White (OR = 2.62, 95% CI [1.27, 5.46], *p* = 0.009) and Hispanic/White (OR = 2.24, 95% CI [1.27, 3.97], *p* = 0.005) matched samples, with no significant matched-sample IL-6 × ethnoracial group interactions.

**Conclusion:**

Plasma IL-6 emerged as a consistent inflammatory correlate of cognitive impairment across Black, Hispanic, and non-Hispanic White older adults. The findings support IL-6 as a potentially generalizable biomarker while highlighting the need for longitudinal studies to clarify its role in depression-related cognitive decline in diverse populations.

## Introduction

Alzheimer’s disease and related dementias (ADRDs) represent a major and growing public health challenge. Black older adults face approximately twice the risk, and Hispanic older adults about 1.5 times the risk, of developing ADRDs compared with non-Hispanic White adults (Alzheimer’s Association, 2020). Research focused on disparities in ADRD prevalence has increasingly recognized stress and inflammation as potential drivers of these disparities (Banks et al., 2006; Letang et al., 2021; Morris et al., 2024). Ethno-racial minorities in the United States experience a confluence of lifestyle and sociological factors that, when combined with certain genetic profiles, may elevate levels of inflammation (Vijendra et al., 2025; Lawrence et al., 2022; Nazmi and Victora, 2007).

Systematic inflammation has emerged as a key contributor to ADRD risk and cognitive aging. Neuroinflammation occurs when immune cells in the brain, particularly microglia and astrocytes, become activated in response to neuronal injury, toxic proteins, or inflammatory signals from elsewhere in the body (Newcombe et al., 2018). Overactivation of these cells leads to the release of pro-inflammatory mediators including cytokines that impair synaptic function and accelerate neuronal loss.

A key inflammatory cytokine implicated in AD is interleukin-6 (IL-6), which has been linked increased is associated with Amyloid-β deposition (Lin et al., 2022), tau hyperphosphorylation (Quintanilla et al., 2004), poorer cognitive performance (Zhao et al., 2024), and reduced hippocampal and cortical volumes (Capogna et al., 2023). IL-6 coordinates innate and adaptive immune responses. Although transient IL-6 activity can be protective, sustained IL-6 trans-signaling is predominantly proinflammatory and can perpetuate chronic immune activation (Tanaka et al., 2014; Rose-John, 2026). Psychological stress may upregulate IL-6 through repeated activation of the sympathetic nervous system and hypothalamic–pituitary–adrenal axis; over time, altered β-adrenergic signaling and reduced glucocorticoid sensitivity can weaken normal anti-inflammatory feedback (Palpatzis et al., 2024; Morgese et al., 2017). In Alzheimer’s disease, sustained IL-6 signaling could contribute mechanistically by promoting microglial and astrocytic activation, vascular and blood–brain barrier dysfunction, and oxidative stress thereby amplifying amyloid- and tau-associated inflammation, synaptic dysfunction, and neuronal injury (Kinney et al., 2018; Wang et al., 2015). However, IL-6 may also be a downstream marker of an already active neurodegenerative process; for example, higher cerebrospinal-fluid IL-6 relates to higher neurofilament light, a marker of axonal injury, in early Alzheimer’s disease (Plantone et al., 2025). Thus, IL-6 is best characterized as both a plausible contributor to inflammatory neurodegeneration and a candidate biomarker, while we need longitudinal and intervention studies before we can consider IL-6 a validated therapeutic target.

While we still do not fully understand the mechanistic role of IL-6 on the generation of dementia pathology, IL-6 consistently confers a greater risk for cognitive decline. A meta-analytic review suggests that serum levels of IL-6 identify the extent of cognitive decline among patients with AD (Lai et al., 2017), and higher IL-6 confers a 1.42-fold increased likelihood of global cognitive decline over 2–7 years of follow-up (Bradburn et al., 2018). Similar associations have been observed in multi-ethnic cohorts, including the Northern Manhattan Study, where higher IL-6 was negatively associated with cognitive function (Mini-Mental State Examination scores) in Hispanic, Black, and non-Hispanic White participants after adjustment for sociodemographic and vascular risk factors (Economos et al., 2013). Determining whether IL-6 levels confer comparable risk across ethno-racial groups may help identify generalizable dementia biomarkers suitable as intervention endpoints.

Sex differences may influence both the development and detection of cognitive impairment. Inflammatory and neuroimmune processes differ by sex, with variation in immune regulation (Klein and Flanagan, 2016) and cytokine responses (Caldwell et al., 2021) potentially influencing vulnerability to cognitive decline (Lutshumba et al., 2023). Additional research has found that women may maintain cognitive performance despite comparable levels of cellular dysfunction (Sundermann et al., 2016). This cognitive resilience can potentially mask early cognitive changes and contribute to delays in clinical detection of impairment.

Differences in inflammatory pathways, particularly levels of IL-6, could explain sex disparities in ADRD. Women particularly of post-menopausal age exhibit greater monocyte expression of IL-6 across the circadian cycle (O’Connor et al., 2007) and show accelerated, more sustained IL-6 increases following acute psychosocial stress (Endrighi et al., 2016) or experimental pain compared with men (Mun et al., 2020). These patterns may contribute to stronger associations between IL-6 and frailty risk observed in older women (Yang et al., 2025), underscoring the need to consider sex as a biological variable in inflammation research. These observations raise the possibility that the association between IL-6 and cognitive impairment may differ by sex, or that sex may interact with race/ethnicity to shape inflammatory risk profiles. However, few studies have explicitly tested whether the relationship between IL-6 and cognitive impairment varies across sex and race/ethnicity groups in diverse aging cohorts.

Depressive symptoms are also robustly linked to late-life cognitive decline and progression to dementia. One hypothesized mechanism involves inflammatory signaling: depression is reliably associated with elevated peripheral IL-6 (Roohi et al., 2021), which may partially transduce depressive burden into accelerated cognitive impairment. However, evidence for IL-6 as a mechanistic bridge remains mixed, and most prior studies have been conducted in predominantly non-Hispanic White samples. Town et al (Town, 2024). found that across various populations, social determinants of health, such as discrimination, ongoing chronic stressors, and inequities in healthcare access, are linked to AD. Furthermore, the negative cognitive impacts of depression tend to be more pronounced in specific racial and ethnic minority groups, who often encounter compounded social and economic challenges that heighten both their depression and cognitive vulnerability (Cheng et al., 2025).

The persistent underrepresentation of racially, ethnically, and gender-diverse groups in Alzheimer’s research creates significant knowledge gaps, emphasizing the urgent need for studies that deliberately include populations at greater risk due to disproportionate adversity and elevated dementia risk (Maestre et al., 2024). Clarifying whether IL-6 represents a shared biological pathway linking depressive symptoms to cognitive impairment across sex and racial/ethnic groups is therefore critical for equitable detection and intervention strategies in diverse aging populations.

In the present study, we examined the interrelationships among depressive symptoms, plasma IL-6, and binary cognitive impairment diagnosis (mild cognitive impairment or dementia versus normal cognition) in the Health and Aging Brain Study–Health Disparities (HABS-HD) cohort (Petersen et al., 2025). We tested whether higher IL-6 was associated with greater odds of cognitive impairment and whether IL-6 mediated the association between depressive symptoms and cognitive impairment. We additionally explored potential moderation by sex and race/ethnicity. By framing IL-6 as a potentially shared inflammatory pathway across diverse older adults, this study aims to clarify whether inflammation represents a common biological mechanism linking depressive symptoms to cognitive risk, independent of racial/ethnic group membership.

## Methods

### Sample demographics

The analytic sample included HABS-HD participants with complete data on cognitive diagnosis, Geriatric Depression Scale (GDS; Montorio and Izal, 1996) score, plasma IL-6, age, education, sex, and race/ethnicity. Cognitive impairment was operationalized as a binary diagnostic outcome, with participants classified as cognitively unimpaired or cognitively impaired. The impaired group included individuals diagnosed with mild cognitive impairment or dementia. The complete-case sample included participants across three racial/ethnic groups: 277 Black participants, 947 Hispanic participants, and 963 White participants. Because the racial/ethnic groups differed in sample size and demographic composition, matched samples were created separately for Black/White and Hispanic/White comparisons to reduce imbalance in key clinical and demographic characteristics.

For the Black/White matched analyses, a 1:1 matched sample was created by matching White participants to Black participants. Participants were matched exactly on sex and cognitive impairment status and balanced on age and years of education. The final Black/White matched sample included 554 participants, with 277 Black participants and 277 White participants. The matched groups had identical distributions of sex and cognitive impairment status. Each racial group included 201 cognitively unimpaired participants and 76 cognitively impaired participants, and 82 females and 195 males in each racial group. After matching, age and education were well balanced across groups; Black participants had a mean age of approximately 62.9 years, White participants had a mean age of approximately 63.7 years, and mean education was nearly identical across groups at approximately 15.2 years.

For the Hispanic/White matched analyses, a separate 1:1 matched sample was created by matching White participants to Hispanic participants. Participants were matched exactly on sex and cognitive impairment status and balanced using calipers for age and education. Although the Hispanic and non-Hispanic White groups were similar in overall sample size, matched analyses were conducted to reduce imbalance in demographic and clinical characteristics that may confound associations among IL-6, depressive symptoms, and cognitive impairment. The final Hispanic/White matched sample included 794 participants, with 397 Hispanic participants and 397 White participants. Each group included 323 cognitively unimpaired participants and 74 cognitively impaired participants, 147 males and 250 females. White participants had a mean age of approximately 64.2 years, and mean education was approximately 13.9 years among Hispanic participants and 14.2 years among White participants.

### Inflammatory cytokines

Peripheral inflammatory biomarkers were measured from HABS-HD plasma and serum samples processed by the Institute for Translational Research (ITR) Biomarker Core laboratory. Proteomic assay preparation was conducted using automated procedures on a customized Hamilton Robotics StarPlus system, with re-aliquoting performed using the Hamilton easyBlood robotic system when necessary (Petersen et al., 2025). These automated procedures were used to improve assay reliability, reduce error and coefficients of variation, and support quality assurance/quality control monitoring.

In the present study, the primary inflammatory marker was log-transformed Simoa plasma IL-6. Secondary inflammatory markers included available Simoa plasma IL-5, IL-10, and TNF-α, as well as MSD serum IL-6, IL-10, and TNF-α where sample coverage permitted. Because circulating cytokine values are typically right-skewed, cytokines were log-transformed prior to analysis.

### Geriatric depression scale

Depressive symptoms were assessed using the Geriatric Depression Scale (GDS) total score (Montorio and Izal, 1996). The GDS is a 15-item measure consisting of yes or no questions indicating presence or absence of a depressive symptom. Higher scores indicate greater depressive symptom burden for a maximum of 15 points.

### Statistical analysis

All statistical analyses were performed using R statistical software (version 4.3). Plasma IL-6 concentrations were log₁₀-transformed before analysis to reduce skewness. Cognitive impairment was modeled as a binary outcome, with mild cognitive impairment or dementia coded as 1 and cognitively unimpaired status coded as 0. All analyses used complete-case data. Statistical significance was evaluated using a two-tailed α level of 0.05. Where multiple cytokines were examined, *p*-values were adjusted using the Benjamini–Hochberg false discovery rate procedure.

### Primary analyses in the full multi-ethnic sample

Primary analyses were conducted in the full multi-ethnic complete-case sample. Binary logistic regression models first examined whether higher log-transformed IL-6 was associated with greater odds of cognitive impairment after adjustment for age, education, sex, ethnoracial group, GDS total score, and APOE ε4 carrier status. Parallel logistic regression models examined the association between GDS total score and cognitive impairment after adjustment for log-transformed IL-6, age, education, sex, ethnoracial group, and APOE ε4 carrier status.

Mediation analyses then tested whether log-transformed IL-6 statistically mediated the association between depressive symptoms and cognitive impairment in the full sample. Linear regression estimated the a-path from GDS total score to log-transformed IL-6. Logistic regression estimated the b-path from log-transformed IL-6 to cognitive impairment while controlling for GDS total score. Models adjusted for age, education, sex, ethnoracial group, and APOE ε4 carrier status. Indirect effects were estimated using the product-of-coefficients approach with Sobel tests.

Moderation analyses evaluated whether the associations of IL-6 and GDS with cognitive impairment differed across ethnoracial groups or by sex. Separate logistic regression models tested IL-6 × ethnoracial group, IL-6 × sex, GDS × ethnoracial group, and GDS × sex interaction terms. Non-Hispanic White participants served as the reference group for ethnoracial comparisons, and male participants served as the reference group for sex comparisons. The joint significance of multi-level ethnoracial interaction terms was evaluated using likelihood-ratio tests. An exploratory model additionally tested the IL-6 × GDS × sex interaction.

### Propensity score-matched sensitivity analyses

To evaluate whether the primary findings remained evident after reducing demographic imbalance across ethnoracial groups, sensitivity analyses were conducted in separate 1:1 propensity score-matched Black/White and Hispanic/White samples. Matching was exact on sex and cognitive impairment status and balanced on age and education. Within each matched sample, logistic regression models repeated the primary analyses examining the associations of log-transformed IL-6 and GDS total score with cognitive impairment. Models adjusted for age, education, sex, ethnoracial group within the matched sample, GDS total score or log-transformed IL-6 as appropriate, and APOE ε4 carrier status. IL-6 × ethnoracial group interaction terms were then tested to determine whether the IL-6–cognitive impairment association differed between groups after demographic balancing. GDS × ethnoracial group interaction terms were also tested within each matched sample to determine whether the association between depressive symptoms and cognitive impairment differed between groups after demographic balancing.

Mediation analyses were also repeated separately in the Black/White and Hispanic/White matched samples using the same covariates and indirect-effect estimation procedures as the full-sample mediation model.

### Group-specific exploratory mediation analyses

Because the relationship among depressive symptoms, inflammation, and cognitive impairment may vary across ethnoracial groups, exploratory mediation analyses were conducted separately among non-Hispanic White, Black, and Hispanic participants. These models tested whether log-transformed IL-6 statistically mediated the association between GDS total score and cognitive impairment within each group. Models adjusted for age, education, sex, and APOE ε4 carrier status. Group-specific findings were interpreted cautiously because differences in statistical significance across subgroups do not necessarily indicate statistically significant between-group differences.

### Secondary cytokine analyses

Secondary logistic regression models evaluated available plasma and serum cytokines to determine whether associations with cognitive impairment were specific to IL-6 or reflected a broader inflammatory profile.

## Results

### Participant characteristics

The full multi-ethnic complete-case sample included 2,173 participants: 959 non-Hispanic White participants, 278 Black participants, and 936 Hispanic participants. The Black/White propensity score-matched sample included 554 participants, with 277 Black and 277 White participants. The Hispanic/White matched sample initially included 794 participants; six participants were excluded from the adjusted analyses because APOE ε4 carrier status was unavailable, resulting in a final analytic sample of 788 participants. Participant characteristics for the full sample are presented in Table 1; matched-sample characteristics are presented in Supplementary Tables S1 and S2.

**Table 1:** Demographic characteristics by ethnoracial group and cognitive impairment status. Full complete-case sample for Simoa plasma IL-6, GDS, APOE ε4, MMSE, and cognitive diagnosis (*N* = 2,173).

Characteristic	White cognitively unimpaired	White cognitively impaired	Black cognitively unimpaired	Black cognitively impaired	Hispanic cognitively unimpaired	Hispanic cognitively impaired
*N*	788	171	202	76	706	230
Male, *n* (%)	316 (40.1%)	103 (60.2%)	57 (28.2%)	25 (32.9%)	239 (33.9%)	90 (39.1%)
Female, *n* (%)	472 (59.9%)	68 (39.8%)	145 (71.8%)	51 (67.1%)	467 (66.1%)	140 (60.9%)
Age, mean (SD)	68.42 (8.32)	70.92 (9.67)	62.09 (6.89)	64.87 (9.28)	62.99 (7.60)	65.40 (8.56)
Years of education, mean (SD)	15.73 (2.53)	15.18 (2.46)	15.44 (2.58)	14.68 (2.41)	9.99 (4.55)	8.88 (4.80)
APOE ε4 carrier, *n* (%)	217 (27.5%)	68 (39.8%)	72 (35.6%)	32 (42.1%)	124 (17.6%)	52 (22.6%)
MMSE total score, mean (SD)	29.18 (1.03)	26.70 (3.60)	28.50 (1.42)	26.55 (3.07)	26.97 (2.66)	23.37 (5.29)
Simoa plasma IL-6, median [IQR]	0.85 [0.55, 1.23]	0.94 [0.68, 1.46]	0.89 [0.61, 1.33]	1.20 [0.77, 1.68]	1.03 [0.69, 1.51]	1.04 [0.74, 1.64]
GDS total score, mean (SD)	4.23 (4.58)	6.49 (6.31)	5.32 (5.22)	6.75 (5.86)	5.90 (5.87)	8.17 (6.70)

Cognitive impairment was defined as MCI or dementia versus cognitively unimpaired status. Race/ethnicity classification prioritized Hispanic ethnicity, followed by non-Hispanic Black and non-Hispanic White classification. IL-6 is shown as raw Simoa plasma IL-6 median [IQR] because cytokine concentrations are typically right-skewed; regression models used log-transformed IL-6. Sex was coded as 0 = male and 1 = female in the source dataset.

### Associations of IL-6 and depressive symptoms with cognitive impairment in the full sample

Higher log-transformed plasma IL-6 was significantly associated with greater odds of cognitive impairment after adjustment for age, education, sex, ethnoracial group, GDS total score, and APOE ε4 carrier status (OR = 1.31, 95% CI [1.12, 1.53], *p* = 0.001). Higher GDS scores were also independently associated with cognitive impairment. Each 1-point increase in GDS total score was associated with approximately 6% greater odds of cognitive impairment after adjustment for IL-6 and the remaining covariates (OR = 1.06, 95% CI [1.04, 1.08], *p* < 0.001). Full multivariable logistic regression results are presented in Table 2.

**Table 2:** Multivariable logistic regression model examining main effects of IL-6, depressive symptoms, and demographic factors on cognitive impairment in the full sample. Full complete-case analytic sample (*N* = 2,173).

Term	Beta	SE	OR	CI Low	CI High	*z*	*p*
Intercept	−3.51	0.57	0.03	0.01	0.09	−6.17	0.000
Black ethnicity (vs. non-Hispanic White)	0.74	0.17	2.09	1.49	2.92	4.29	0.000
Hispanic ethnicity (vs. non-Hispanic White)	0.33	0.16	1.39	1.02	1.89	2.10	0.036
IL-6	0.27	0.08	1.31	1.12	1.53	3.43	0.001
GDS	0.06	0.01	1.06	1.04	1.08	6.45	0.000
Age	0.03	0.01	1.03	1.02	1.05	5.06	0.000
Education	−0.03	0.02	0.97	0.94	0.99	−2.31	0.021
Female sex	−0.52	0.11	0.60	0.48	0.74	−4.67	0.000
APOE ε4 carrier	0.47	0.12	1.60	1.26	2.02	3.92	0.000

The outcome was cognitive impairment status. IL-6 was log-transformed. OR = odds ratio; CI = 95% confidence interval; GDS = Geriatric Depression Scale; APOE4 = APOE ε4 carrier status. Sex was coded as 0 = male and 1 = female; female sex is shown relative to male sex.

### Mediation analysis in the full sample

Mediation analysis tested whether plasma IL-6 statistically mediated the association between depressive symptoms and cognitive impairment. Higher GDS scores were significantly associated with higher log-transformed IL-6 concentrations (a-path: *β* = 0.009, SE = 0.003, *p* < 0.001). Higher IL-6 remained significantly associated with cognitive impairment after adjustment for GDS total score and the remaining covariates (b-path: OR = 1.31, 95% CI [1.12, 1.53], *p* = 0.001). The indirect effect of GDS on cognitive impairment through IL-6 was statistically significant (indirect effect = 0.002, Sobel *z* = 2.46, *p* = 0.014). Full-sample, matched-sample, and group-specific mediation results are summarized in Table 3.

**Table 3:** Mediation models examining GDS → IL-6 → cognitive impairment. Indirect effects are reported using the product-of-coefficients approach with Sobel tests.

Analysis	*N*	a-path beta (GDS→IL-6)	a-path SE	a-path *p*	b-path beta (IL-6 → CogImp)	b-path OR	b-path CI Low	b-path CI High	b-path *p*	Indirect effect (a × b)	Sobel SE	Sobel *z*	Sobel *p*
Full sample	2,173	0.009	0.003	0.000	0.272	1.313	1.124	1.534	0.001	0.002	0.001	2.461	0.014
Black/White matched	554	0.007	0.005	0.136	0.420	1.522	1.110	2.088	0.009	0.003	0.002	1.296	0.195
Hispanic/White matched	788	0.011	0.005	0.021	0.352	1.422	1.112	1.819	0.005	0.004	0.002	1.786	0.074
White only	959	0.014	0.004	0.001	0.284	1.328	1.031	1.710	0.028	0.004	0.002	1.818	0.069
Black only	278	0.007	0.006	0.248	0.631	1.879	1.140	3.096	0.013	0.005	0.004	1.048	0.295
Hispanic only	936	0.005	0.004	0.162	0.151	1.163	0.931	1.453	0.184	0.001	0.001	0.963	0.336

Group-specific mediation models are exploratory; differences in subgroup significance do not establish statistically significant between-group differences. GDS = Geriatric Depression Scale; IL-6 = interleukin-6; CogImp = cognitive impairment; OR = odds ratio; CI = 95% confidence interval.

### Moderation by ethnoracial group and sex in the full sample

The association between IL-6 and cognitive impairment did not differ significantly across ethnoracial groups (likelihood-ratio χ^2^ = 3.52, df = 2, *p* = 0.172). Group-specific estimates showed significant positive associations among non-Hispanic White participants (OR = 2.03, 95% CI [1.17, 3.58], *p* = 0.012) and Black participants (OR = 4.38, 95% CI [1.46, 13.33], *p* = 0.008). The association was positive but did not reach statistical significance among Hispanic participants (OR = 1.44, 95% CI [0.87, 2.39], *p* = 0.168). Neither the IL-6 × Black interaction term (OR = 2.16, 95% CI [0.64, 7.28], *p* = 0.223) nor the IL-6 × Hispanic interaction term (OR = 0.71, 95% CI [0.33, 1.52], *p* = 0.358) was statistically significant (Supplementary Table S3; Figure 1).

**Figure 1:**
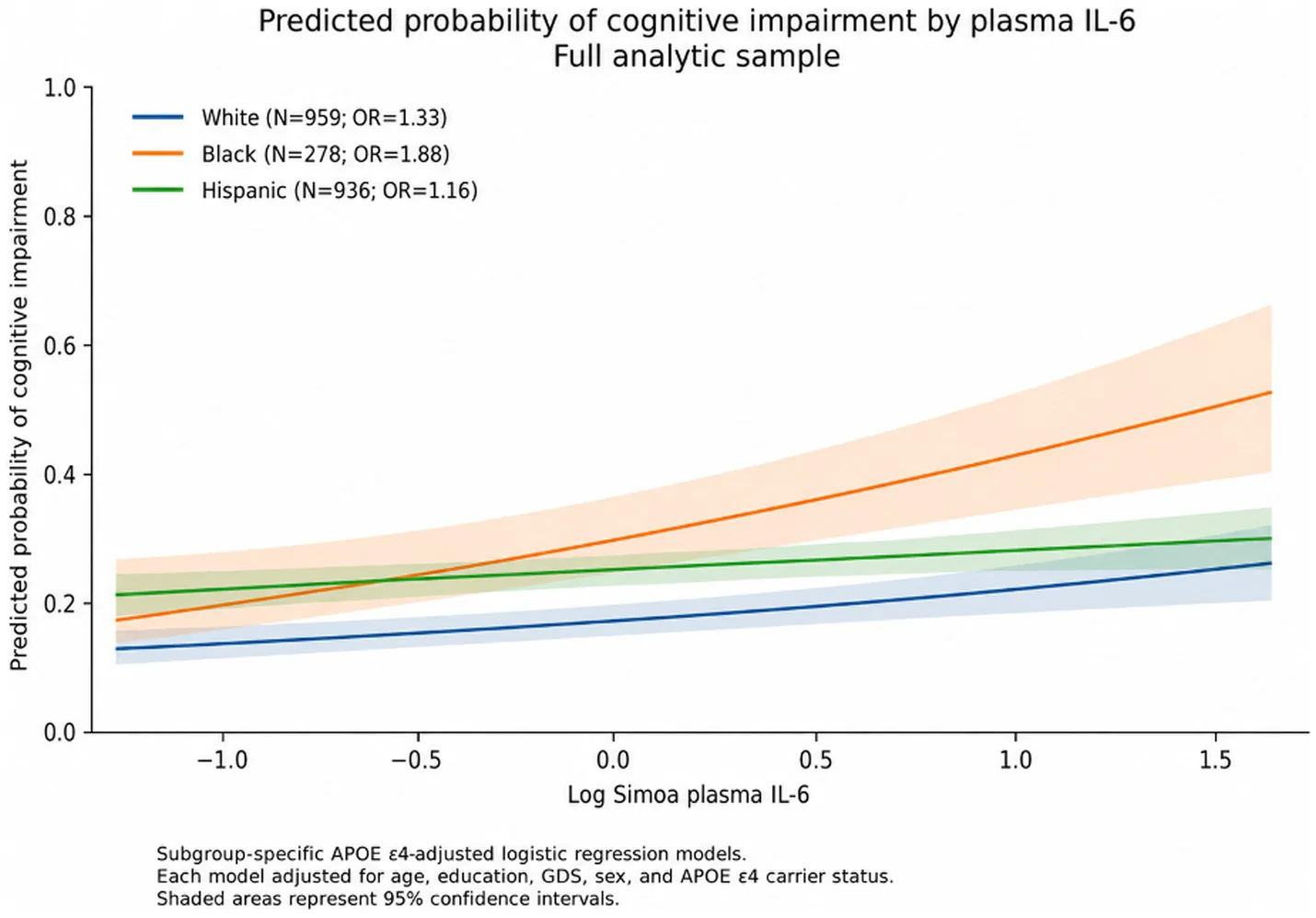
Association between plasma IL-6 and predicted probability of cognitive impairment across ethnoracial groups. Shaded regions indicate 95% confidence intervals.

The association between GDS scores and cognitive impairment also did not differ significantly across ethnoracial groups (likelihood-ratio χ^2^ = 2.91, df = 2, *p* = 0.233; Supplementary Table S4). The IL-6 × sex interaction was not statistically significant (OR = 0.87, 95% CI [0.43, 1.77], *p* = 0.685; Supplementary Table S5). The GDS × sex interaction approached but did not reach statistical significance (OR = 0.97, 95% CI [0.93, 1.00], *p* = 0.056; Supplementary Table S6). In an exploratory model, the IL-6 × GDS × sex interaction was not statistically significant (OR = 0.95, 95% CI [0.83, 1.07], *p* = 0.406; Supplementary Table S7; Figure 2).

**Figure 2:**
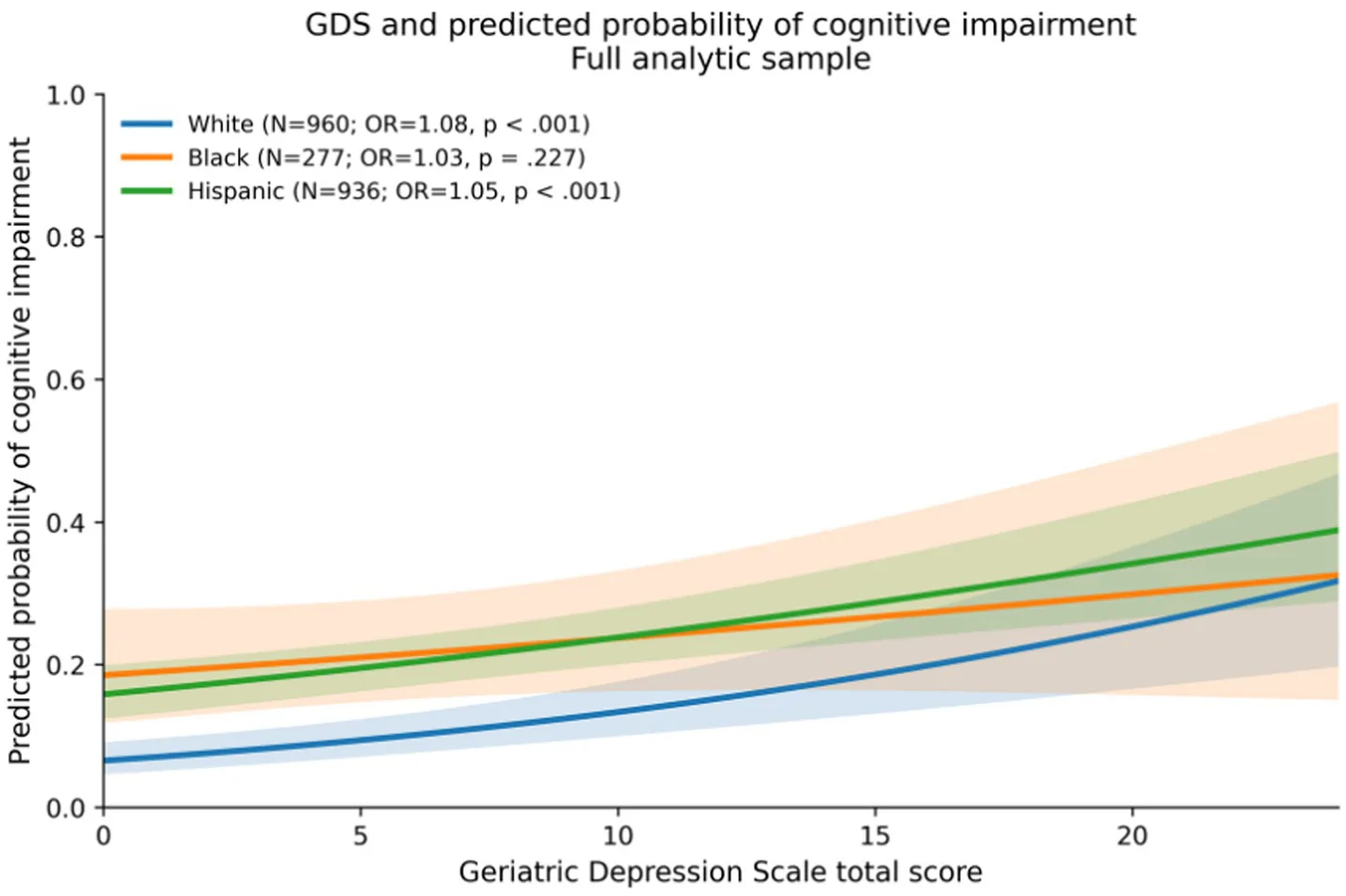
Depressive symptoms and predicted probability of cognitive impairment by ethno-racial group. Shaded area represents 95% CI. GDS = Geriatric Depression Scale.

### Matched-sample sensitivity analyses

In the Black/White matched sample (*N* = 554), higher log₁₀-transformed IL-6 was associated with greater odds of cognitive impairment after adjustment for age, education, sex, ethnoracial group, GDS total score, and APOE ε4 carrier status (OR = 2.62, 95% CI [1.27, 5.46], *p* = 0.009). Higher GDS scores were also associated with cognitive impairment (OR = 1.04, 95% CI [1.01, 1.08], *p* = 0.019). Neither the IL-6 × ethnoracial group interaction (OR = 1.86, 95% CI [0.43, 8.18], *p* = 0.404) nor the GDS × ethnoracial group interaction (OR = 0.98, 95% CI [0.92, 1.05], *p* = 0.598) was statistically significant (Supplementary Table S9, Panel A).

In the Hispanic/White matched sample (*N* = 788), higher log₁₀-transformed IL-6 was associated with greater odds of cognitive impairment (OR = 2.24, 95% CI [1.27, 3.97], *p* = 0.005). Higher GDS scores were also associated with cognitive impairment (OR = 1.06, 95% CI [1.03, 1.10], *p* < 0.001). Neither the IL-6 × ethnoracial group interaction (OR = 0.91, 95% CI [0.30, 2.79], *p* = 0.859) nor the GDS × ethnoracial group interaction (OR = 0.96, 95% CI [0.90, 1.02], *p* = 0.168) was statistically significant (Supplementary Table S9, Panel B).

### Matched-sample mediation analyses

In the Black/White matched sample, the association between GDS total score and log-transformed IL-6 was not statistically significant (a-path: *β* = 0.007, SE = 0.005, *p* = 0.136), although IL-6 remained significantly associated with cognitive impairment after adjustment for GDS total score (b-path: OR = 1.52, 95% CI [1.11, 2.09], *p* = 0.009). The indirect effect through IL-6 was not statistically significant (indirect effect = 0.003, Sobel *z* = 1.30, *p* = 0.195; Table 3).

In the Hispanic/White matched sample, higher GDS scores were significantly associated with higher log-transformed IL-6 concentrations (a-path: *β* = 0.011, SE = 0.005, *p* = 0.021), and IL-6 remained significantly associated with cognitive impairment after adjustment for GDS total score (b-path: OR = 1.42, 95% CI [1.11, 1.82], *p* = 0.005). The indirect effect approached but did not reach statistical significance (indirect effect = 0.004, Sobel *z* = 1.79, *p* = 0.074; Table 3).

### Group-specific exploratory mediation analyses

Exploratory mediation models were conducted separately among non-Hispanic White, Black, and Hispanic participants. Among non-Hispanic White participants, higher GDS scores were associated with higher IL-6 concentrations (a-path: *β* = 0.014, *p* = 0.001), and IL-6 remained associated with cognitive impairment after adjustment for GDS total score (b-path: OR = 1.33, 95% CI [1.03, 1.71], *p* = 0.028). The indirect effect approached but did not reach statistical significance (indirect effect = 0.004, Sobel *p* = 0.069; Table 3).

Among Black participants, GDS scores were not significantly associated with IL-6 concentrations (a-path: *β* = 0.007, *p* = 0.248), although IL-6 remained associated with cognitive impairment after adjustment for GDS total score (b-path: OR = 1.88, 95% CI [1.14, 3.10], *p* = 0.013). The indirect effect was not statistically significant (indirect effect = 0.005, Sobel *p* = 0.295; Table 3).

Among Hispanic participants, neither the association between GDS scores and IL-6 concentrations (a-path: *β* = 0.005, *p* = 0.162) nor the association between IL-6 and cognitive impairment after adjustment for GDS total score (b-path: OR = 1.16, 95% CI [0.93, 1.45], *p* = 0.184) reached statistical significance. The indirect effect was also not statistically significant (indirect effect = 0.001, Sobel *p* = 0.336; Table 3).

These group-specific mediation models were exploratory. Differences in the statistical significance of subgroup-specific indirect effects should not be interpreted as evidence of statistically significant between-group differences.

### Secondary cytokine analyses

Secondary analyses examined whether associations with cognitive impairment were specific to plasma IL-6 or reflected a broader inflammatory profile. Separate logistic regression models tested associations between each log₁₀-transformed cytokine and cognitive impairment after adjustment for age, education, sex, ethnoracial group, Geriatric Depression Scale total score, and APOE ε4 carrier status. Among the secondary cytokines, higher plasma IL-5 was associated with lower odds of cognitive impairment (OR = 0.64, 95% CI [0.46, 0.89], *p* = 0.007) and remained significant after false discovery rate correction (FDR-adjusted *p* = 0.043). IL-10 and TNF-α were not significantly associated with cognitive impairment. Full results are presented in Supplementary Table S8.

### Sensitivity analysis: chronic stress

In an exploratory sensitivity analysis, chronic stress data were available for 2,168 participants with complete IL-6, cognitive diagnosis, depressive symptom, and demographic data. Higher chronic stress was associated with higher log₁₀-transformed plasma IL-6 after adjustment for age, education, sex, ethnoracial group, and GDS total score (*β* = 0.021 per 5-point increase, 95% CI [0.011, 0.031], *p* < 0.001). However, chronic stress was not independently associated with cognitive impairment after adjustment for IL-6 and the remaining covariates (OR = 0.96, 95% CI [0.88, 1.04], *p* = 0.330). The association between IL-6 and cognitive impairment remained significant and was materially unchanged after chronic stress was added to the model (OR = 1.79, 95% CI [1.26, 2.56], *p* = 0.001), suggesting that chronic stress did not account for the observed IL-6 association. Full results from the chronic-stress sensitivity analysis are presented in Supplementary Table S10.

## Discussion

In this study we explored associations among IL-6, geriatric depression scores, and cognitive impairment in a diverse sample of older adults. Across analyses, plasma IL-6 emerged as the most consistent inflammatory marker associated with cognitive impairment. In the full complete-case sample, higher IL-6 was associated with increased odds of MCI/dementia, and formal interaction testing did not provide evidence that this association differed significantly across ethnoracial groups. The overall IL-6 × ethnoracial group interaction was not significant, suggesting that IL-6 may reflect a shared inflammatory correlate of cognitive impairment rather than a pathway specific to one ethnoracial group. This finding persisted in the propensity score-matched subsamples, where we minimized demographic confounding through exact matching on sex and cognitive status. In both the Black/White and Hispanic/White matched samples, higher log-transformed IL-6 remained significantly associated with greater odds of cognitive impairment. IL-6 appears to be a shared inflammatory correlate of cognitive impairment across the sample, rather than a clearly group-specific marker. However, the Hispanic-specific estimate was weaker and nonsignificant, which may reflect heterogeneity within the Hispanic sample or other demographic, vascular, metabolic, or sociocultural factors. Our results extend prior multi-ethnic work (Economos et al., 2013; Leonardo and Fregni, 2023), which similarly reported IL-6–cognition associations without strong race/ethnic moderation, and align with meta-analytic evidence linking elevated IL-6 to global cognitive decline.

At higher IL-6 concentrations, the modeled probability of cognitive impairment appeared numerically greater among Black participants than among non-Hispanic White or Hispanic participants. This pattern was reflected in a larger group-specific effect estimate among Black participants; however, the IL-6-by-ethnoracial-group interaction was not statistically significant, and the estimate in the smaller Black subgroup had a wide confidence interval. Thus, the observed pattern should not be interpreted as evidence of a definitive between-group difference. One possibility is that elevated IL-6 captures a greater cumulative burden of vascular, metabolic, environmental, and chronic stress-related exposures among Black participants, which may jointly increase vulnerability to cognitive impairment. Future studies incorporating cardiometabolic risk, cerebrovascular imaging, social determinants of health, and longitudinal inflammatory measures will be needed to evaluate these potential pathways.

Depressive symptoms were also consistently related to cognitive impairment in both the full and matched samples, replicating well-established links between late-life depression and increased dementia risk. Meta-analyses of community-based prospective studies have shown that late-life depression is associated with approximately 1.85-fold higher risk of all-cause dementia and 1.65-fold higher risk of AD (Diniz et al., 2013). Mediation analyses, however, provided only modest support for IL-6 as a mechanistic bridge. In the Hispanic/White matched sample, higher GDS scores were significantly associated with higher plasma IL-6, producing a trend-level indirect effect on cognitive impairment. In contrast, the GDS to IL-6 path was not significant in the Black/White matched sample, resulting in a nonsignificant indirect effect. Collectively, these results suggest that IL-6 may partially transduce depressive symptoms into cognitive risk, with possible ethnic variation in the strength of this pathway. The absence of a statistically significant association between depressive symptoms and IL-6 among Black participants was unlikely to be attributable to lower mean depressive-symptom levels, which were higher than those observed among non-Hispanic White participants. Instead, the finding may reflect the smaller Black subsample, reduced statistical precision, and greater heterogeneity in the social, behavioral, and cardiometabolic determinants of systemic inflammation. Additionally, ethnoracial differences in the expression or measurement of depressive symptoms may weaken associations between total GDS scores and circulating IL-6. Because subgroup mediation models were exploratory and cross-sectional, these explanations require direct evaluation in larger longitudinal samples. This pattern is consistent with emerging evidence that inflammatory mechanisms interact with vascular (Raz et al., 2016), metabolic (Tian et al., 2025), and psychosocial factors (McDermott et al., 2019; Chen et al., 2026) that themselves vary by race/ethnicity, limiting the explanatory power of any single biomarker (Brain et al., 2025; Wilson et al., 2016).

Secondary cytokine analyses suggested that the relationship between peripheral inflammation and cognitive impairment was not explained by a generalized elevation across the broader cytokine panel. Instead, the pattern appeared more selective. IL-6 showed the clearest positive association with cognitive impairment, consistent with prior work linking IL-6 signaling to cognitive decline, AD pathophysiology, and systemic inflammatory or metabolic burden. In contrast, IL-10 and TNF-α did not show significant associations with cognitive impairment in the fully adjusted models despite their frequent discussion in the AD and neuroinflammation literature (Magalhães et al., 2017; Plantone et al., 2023). This lack of association may reflect the heterogeneity of peripheral cytokine findings across studies, differences in disease stage, comorbidity burden, assay sensitivity, or the possibility that these markers do not capture the same chronic inflammatory pathway indexed by IL-6 in this cohort. Notably, IL-5 showed an inverse association with cognitive impairment, such that higher IL-5 corresponded to lower odds of impairment. Unlike IL-6, IL-5 does not consistently positively or negatively correlate with cognitive performance (Chen et al., 2018) or confer a greater risk for cognitive impairment (Swardfager et al., 2010). IL-5 may reflect a distinct immune-regulatory or type 2 inflammatory pathway whose relationship to cognition differs from canonical pro-inflammatory markers. Together, these findings support a targeted interpretation of IL-6 as the most consistent inflammatory correlate of cognitive impairment in our analyses, while also highlighting IL-5 as an exploratory signal warranting replication in longitudinal and mechanistically informed studies.

Strengths of the study include the use of rigorously matched subsamples from a large, community-based diverse cohort, the evaluation of multiple cytokines across two assay platforms, and formal testing of race/ethnic moderation and mediation. The propensity matching approach effectively balanced key confounders, increasing confidence that the observed IL-6 associations are not artifacts of demographic imbalance.

We note several limitations including the lack of longitudinal data and unmeasured potential confounds. Although our models are consistent with a depression–inflammation–cognition pathway, the cross-sectional design precludes conclusions about temporal ordering of depressive symptoms, IL-6, and cognitive impairment. The literature surrounding the mechanistic role of Il-6 in the development of dementia is mixed, with some studies reporting a modest correlation, and others suggestion a causal relationship (Lai et al., 2017; Bradburn et al., 2018). A genetic study identified the individuals predisposed to higher circulating IL-6 levels may be at greater risk for dementia (Charisis et al., 2025). Given the proinflammatory effects of sustained IL-6 signaling and emerging experimental and genetic evidence linking IL-6 pathways to Alzheimer’s disease pathogenesis (Lyra e Silva et al., 2021), IL-6 represents a potential therapeutic target for inflammation-associated cognitive impairment. Nevertheless, the present cross-sectional findings cannot distinguish a causal contribution from a downstream biomarker response. Longitudinal analyses would enable us to determine whether IL-6 precedes cognitive decline, reflects broader disease burden, or participates in a bidirectional pathway linking mood and cognition. Although we adjusted for APOE ε4, unmeasured confounders such as vascular risk, physical activity, or medication use may have influenced results. Depressive symptoms were assessed via self-report (i.e., GDS), which may underestimate symptoms in some cultural groups. Finally, although the matched samples were well balanced, residual differences in unmeasured socioeconomic or health variables could remain.

These findings support the conceptualization of circulating IL-6 as a shared inflammatory risk marker for cognitive impairment that operates similarly across Black, Hispanic, and non-Hispanic White older adults. This has important implications for equitable biomarker development and precision prevention strategies: interventions targeting IL-6 (e.g., lifestyle modification, anti-inflammatory therapies, or depression management) could benefit diverse populations without requiring race/ethnicity-specific tailoring. As the population becomes more aware of the importance of brain health preservation (Siette et al., 2025; Avan and Hachinski, 2024), IL-6 shows promise as a scalable, generalizable biomarker for identifying midlife adults with inflammation-related concerns about brain health. Future longitudinal analyses within HABS-HD and other diverse cohorts should examine whether baseline IL-6 predicts incident cognitive decline and whether changes in IL-6 mediate treatment effects on cognition. Such work will be critical for translating these biomarker associations into actionable strategies to reduce dementia burden across all racial/ethnic groups.

## Data Availability

Publicly available datasets were analyzed in this study. This data can be found here: https://healthandagingbrainstudy.org/.
