# Supplementary material for: Plasma IL-6 as a shared inflammatory marker of cognitive impairment across racially and ethnically diverse older adults: matched analyses from the HABS-HD study
Source: Front Dement. 2026 Oct 14;5:1930196. doi: 10.3389/frdem.2026.1930196 (PMC13713380; doi:10.3389/frdem.2026.1930196)
Supplement: Supplementary file 1 [file Table_1.docx]

**HABS-HD Manuscript Tables and Supplemental Materials**

# Supplemental Tables

**Supplemental Table S1. Demographic characteristics of the Black-White matched analytic sample by cognitive impairment status**

*Matched sample for Simoa plasma IL-6, GDS, APOE ε4, MMSE, and cognitive diagnosis (N = 554)*

| **Characteristic** | **Black Cognitively unimpaired** | **Black Cognitively impaired** | **White Cognitively unimpaired** | **White Cognitively impaired** |
| --- | --- | --- | --- | --- |
| N | 201 | 76 | 201 | 76 |
| Male, n (%) | 57 (28.4%) | 25 (32.9%) | 57 (28.4%) | 25 (32.9%) |
| Female, n (%) | 144 (71.6%) | 51 (67.1%) | 144 (71.6%) | 51 (67.1%) |
| Age, mean (SD) | 62.10 (6.90) | 64.87 (9.28) | 62.65 (6.67) | 66.62 (8.05) |
| Years of education, mean (SD) | 15.44 (2.58) | 14.68 (2.41) | 15.42 (2.46) | 14.70 (2.34) |
| MMSE total score, mean (SD) | 28.50 (1.43) | 26.55 (3.07) | 29.31 (0.89) | 26.66 (4.00) |
| GDS total score, mean (SD) | 5.34 (5.22) | 6.75 (5.86) | 5.50 (5.55) | 7.38 (6.40) |
| APOE ε4 carrier, n (%) | 71 (35.3%) | 32 (42.1%) | 50 (24.9%) | 33 (43.4%) |
| IL-6, median [IQR] | 0.89 [0.61, 1.33] | 1.20 [0.77, 1.68] | 0.82 [0.52, 1.21] | 0.89 [0.64, 1.48] |

**Note.** IL-6 is presented as median [IQR] because cytokine values are typically right-skewed. Other continuous variables are presented as mean (SD). Sex was coded as 0 = male and 1 = female in the source dataset.

**Supplemental Table S2. Demographic characteristics of the Hispanic-White matched analytic sample by cognitive impairment status**

*Complete-case matched analytic sample used for adjusted models (N = 788)*

| **Characteristic** | **White Cognitively unimpaired** | **White Cognitively impaired** | **Hispanic Cognitively unimpaired** | **Hispanic Cognitively impaired** |
| --- | --- | --- | --- | --- |
| N | 322 | 74 | 319 | 72 |
| Male, n (%) | 112 (34.8%) | 35 (47.3%) | 112 (35.1%) | 34 (47.2%) |
| Female, n (%) | 210 (65.2%) | 39 (52.7%) | 207 (64.9%) | 38 (52.8%) |
| Age, mean (SD) | 63.84 (7.54) | 65.84 (7.07) | 63.03 (7.46) | 65.82 (7.12) |
| Years of education, mean (SD) | 14.20 (2.45) | 14.00 (2.23) | 13.93 (2.59) | 13.72 (2.37) |
| APOE ε4 carrier, n (%) | 90 (28.0%) | 29 (39.2%) | 55 (17.2%) | 12 (16.7%) |
| GDS total score, mean (SD) | 4.83 (5.02) | 7.41 (6.66) | 4.86 (5.16) | 6.06 (5.82) |
| IL-6, median [IQR] | 0.86 [0.55, 1.23] | 1.02 [0.73, 1.51] | 0.99 [0.65, 1.47] | 1.26 [0.81, 2.02] |

**Note.** Cognitive impairment was defined as MCI or dementia versus cognitively unimpaired status. IL-6 is presented as median [IQR] because cytokine values are typically right-skewed. Other continuous variables are presented as mean (SD). Sex was coded as 0 = male and 1 = female in the source dataset. The stored Hispanic/White matched set contained 794 participants before complete-case filtering; six participants were excluded because APOE ε4 carrier status was unavailable, yielding N = 788 for adjusted analyses. MMSE is not shown because it was not available for this matched table in the analytic output used to generate it.

**Supplemental Table S3. Multivariable logistic regression model examining the interaction between IL-6 and ethnoracial group on cognitive impairment in the full sample**

*Supplemental logistic regression models from the full complete-case analytic sample.*

| **Term** | **Beta** | **SE** | **OR** | **CI Low** | **CI High** | **z** | **p** |
| --- | --- | --- | --- | --- | --- | --- | --- |
| Intercept | -3.55 | 0.57 | 0.03 | 0.01 | 0.09 | -6.22 | <.001 |
| Black ethnicity (vs. non-Hispanic White) | 0.72 | 0.17 | 2.06 | 1.47 | 2.90 | 4.17 | <.001 |
| Hispanic ethnicity (vs. non-Hispanic White) | 0.36 | 0.16 | 1.43 | 1.05 | 1.95 | 2.27 | .023 |
| Log10 IL-6 | 0.71 | 0.28 | 2.03 | 1.17 | 3.58 | 2.51 | .012 |
| Log10 IL-6 × Black ethnicity | 0.76 | 0.62 | 2.16 | 0.64 | 7.28 | 1.22 | .223 |
| Log10 IL-6 × Hispanic ethnicity | -0.35 | 0.39 | 0.71 | 0.33 | 1.52 | -0.92 | .358 |
| GDS | 0.06 | 0.01 | 1.06 | 1.04 | 1.08 | 6.41 | <.001 |
| Age | 0.03 | 0.01 | 1.03 | 1.02 | 1.05 | 5.09 | <.001 |
| Education | -0.03 | 0.02 | 0.97 | 0.94 | 1.00 | -2.22 | .027 |
| Female sex | -0.52 | 0.11 | 0.59 | 0.48 | 0.74 | -4.71 | <.001 |
| APOE ε4 carrier | 0.46 | 0.12 | 1.59 | 1.26 | 2.01 | 3.86 | <.001 |

**Note.** The outcome was cognitive impairment status. IL-6 was log10-transformed; ORs for IL-6 terms reflect a 10-fold increase in plasma IL-6 concentration. OR = odds ratio; CI = 95% confidence interval; GDS = Geriatric Depression Scale; APOE4 = APOE ε4 carrier status. Sex was coded as 0 = male and 1 = female; female sex is shown relative to male sex.

**Supplemental Table S4. Multivariable logistic regression model examining the interaction between depressive symptoms and ethnoracial group on cognitive impairment in the full sample**

*Supplemental logistic regression models from the full complete-case analytic sample.*

| **Term** | **Beta** | **SE** | **OR** | **CI Low** | **CI High** | **z** | **p** |
| --- | --- | --- | --- | --- | --- | --- | --- |
| Intercept | -3.69 | 0.58 | 0.03 | 0.01 | 0.08 | -6.37 | <.001 |
| Black ethnicity (vs. non-Hispanic White) | 0.98 | 0.25 | 2.66 | 1.64 | 4.31 | 3.97 | <.001 |
| Hispanic ethnicity (vs. non-Hispanic White) | 0.51 | 0.20 | 1.66 | 1.12 | 2.46 | 2.55 | .011 |
| GDS | 0.08 | 0.02 | 1.08 | 1.05 | 1.12 | 5.16 | <.001 |
| GDS × Black ethnicity | -0.04 | 0.03 | 0.96 | 0.91 | 1.02 | -1.38 | .168 |
| GDS × Hispanic ethnicity | -0.03 | 0.02 | 0.97 | 0.94 | 1.01 | -1.49 | .136 |
| Log10 IL-6 | 0.62 | 0.18 | 1.87 | 1.31 | 2.67 | 3.39 | .001 |
| Age | 0.03 | 0.01 | 1.04 | 1.02 | 1.05 | 5.18 | <.001 |
| Education | -0.04 | 0.02 | 0.97 | 0.94 | 0.99 | -2.37 | .018 |
| Female sex | -0.51 | 0.11 | 0.60 | 0.48 | 0.74 | -4.63 | <.001 |
| APOE ε4 carrier | 0.47 | 0.12 | 1.60 | 1.27 | 2.03 | 3.94 | <.001 |

**Note.** The outcome was cognitive impairment status. IL-6 was log10-transformed; ORs for IL-6 terms reflect a 10-fold increase in plasma IL-6 concentration. OR = odds ratio; CI = 95% confidence interval; GDS = Geriatric Depression Scale; APOE4 = APOE ε4 carrier status. Sex was coded as 0 = male and 1 = female; female sex is shown relative to male sex.

**Supplemental Table S5. Multivariable logistic regression model examining the interaction between IL-6 and sex on cognitive impairment in the full sample**

*Supplemental logistic regression models from the full complete-case analytic sample.*

| **Term** | **Beta** | **SE** | **OR** | **CI Low** | **CI High** | **z** | **p** |
| --- | --- | --- | --- | --- | --- | --- | --- |
| Intercept | -3.52 | 0.57 | 0.03 | 0.01 | 0.09 | -6.18 | <.001 |
| Black ethnicity (vs. non-Hispanic White) | 0.74 | 0.17 | 2.10 | 1.50 | 2.93 | 4.31 | <.001 |
| Hispanic ethnicity (vs. non-Hispanic White) | 0.33 | 0.16 | 1.39 | 1.02 | 1.90 | 2.11 | .035 |
| Log10 IL-6 | 0.69 | 0.25 | 1.99 | 1.22 | 3.28 | 2.74 | .006 |
| Female sex | -0.51 | 0.11 | 0.60 | 0.48 | 0.75 | -4.61 | <.001 |
| Log10 IL-6 × Female sex | -0.14 | 0.37 | 0.87 | 0.43 | 1.77 | -0.41 | .685 |
| GDS | 0.06 | 0.01 | 1.06 | 1.04 | 1.08 | 6.46 | <.001 |
| Age | 0.03 | 0.01 | 1.03 | 1.02 | 1.05 | 5.06 | <.001 |
| Education | -0.03 | 0.02 | 0.97 | 0.94 | 0.99 | -2.31 | .021 |
| APOE ε4 carrier | 0.47 | 0.12 | 1.60 | 1.26 | 2.02 | 3.92 | <.001 |

**Note.** The outcome was cognitive impairment status. IL-6 was log10-transformed; ORs for IL-6 terms reflect a 10-fold increase in plasma IL-6 concentration. OR = odds ratio; CI = 95% confidence interval; GDS = Geriatric Depression Scale; APOE4 = APOE ε4 carrier status. Sex was coded as 0 = male and 1 = female; female sex is shown relative to male sex.

**Supplemental Table S6. Multivariable logistic regression model examining the interaction between depressive symptoms and sex on cognitive impairment in the full sample**

*Supplemental logistic regression models from the full complete-case analytic sample.*

| **Term** | **Beta** | **SE** | **OR** | **CI Low** | **CI High** | **z** | **p** |
| --- | --- | --- | --- | --- | --- | --- | --- |
| Intercept | -3.67 | 0.58 | 0.03 | 0.01 | 0.08 | -6.35 | <.001 |
| Black ethnicity (vs. non-Hispanic White) | 0.74 | 0.17 | 2.10 | 1.50 | 2.93 | 4.32 | <.001 |
| Hispanic ethnicity (vs. non-Hispanic White) | 0.34 | 0.16 | 1.40 | 1.03 | 1.91 | 2.13 | .033 |
| GDS | 0.08 | 0.01 | 1.08 | 1.05 | 1.11 | 5.52 | <.001 |
| Female sex | -0.30 | 0.16 | 0.74 | 0.54 | 1.02 | -1.85 | .065 |
| GDS × Female sex | -0.03 | 0.02 | 0.97 | 0.93 | 1.00 | -1.91 | .056 |
| Log10 IL-6 | 0.64 | 0.18 | 1.90 | 1.33 | 2.71 | 3.50 | <.001 |
| Age | 0.03 | 0.01 | 1.03 | 1.02 | 1.05 | 5.09 | <.001 |
| Education | -0.03 | 0.02 | 0.97 | 0.94 | 1.00 | -2.28 | .022 |
| APOE ε4 carrier | 0.46 | 0.12 | 1.59 | 1.25 | 2.01 | 3.84 | <.001 |

**Note.** The outcome was cognitive impairment status. IL-6 was log10-transformed; ORs for IL-6 terms reflect a 10-fold increase in plasma IL-6 concentration. OR = odds ratio; CI = 95% confidence interval; GDS = Geriatric Depression Scale; APOE4 = APOE ε4 carrier status. Sex was coded as 0 = male and 1 = female; female sex is shown relative to male sex.

**Supplemental Table S7. Multivariable logistic regression model examining the three-way interaction among IL-6, depressive symptoms, and sex on cognitive impairment in the full sample**

*Supplemental logistic regression models from the full complete-case analytic sample.*

| **Term** | **Beta** | **SE** | **OR** | **CI Low** | **CI High** | **z** | **p** |
| --- | --- | --- | --- | --- | --- | --- | --- |
| Intercept | -3.65 | 0.58 | 0.03 | 0.01 | 0.08 | -6.32 | <.001 |
| Black ethnicity (vs. non-Hispanic White) | 0.74 | 0.17 | 2.09 | 1.50 | 2.93 | 4.31 | <.001 |
| Hispanic ethnicity (vs. non-Hispanic White) | 0.34 | 0.16 | 1.40 | 1.03 | 1.91 | 2.13 | .033 |
| Log10 IL-6 | 0.32 | 0.39 | 1.38 | 0.63 | 2.99 | 0.80 | .425 |
| GDS | 0.08 | 0.01 | 1.08 | 1.05 | 1.12 | 5.52 | <.001 |
| Log10 IL-6 × GDS | 0.07 | 0.05 | 1.07 | 0.95 | 1.17 | 1.24 | .214 |
| Female sex | -0.28 | 0.16 | 0.75 | 0.55 | 1.03 | -1.76 | .078 |
| Log10 IL-6 × Female sex | 0.21 | 0.53 | 1.23 | 0.43 | 3.48 | 0.37 | .710 |
| GDS × Female sex | -0.04 | 0.02 | 0.96 | 0.93 | 1.00 | -1.95 | .051 |
| Log10 IL-6 × GDS × Female sex | -0.05 | 0.07 | 0.95 | 0.83 | 1.07 | -0.83 | .406 |
| Age | 0.03 | 0.01 | 1.03 | 1.02 | 1.05 | 5.07 | <.001 |
| Education | -0.04 | 0.02 | 0.96 | 0.94 | 0.99 | -2.36 | .018 |
| APOE ε4 carrier | 0.47 | 0.12 | 1.60 | 1.26 | 2.03 | 3.91 | <.001 |

**Note.** The outcome was cognitive impairment status. IL-6 was log10-transformed; ORs for IL-6 terms reflect a 10-fold increase in plasma IL-6 concentration. OR = odds ratio; CI = 95% confidence interval; GDS = Geriatric Depression Scale; APOE4 = APOE ε4 carrier status. Sex was coded as 0 = male and 1 = female; female sex is shown relative to male sex.

**Supplemental Table S8. Associations between inflammatory cytokines and cognitive impairment**

*Separate covariate-adjusted logistic regression models tested each log10-transformed cytokine as a predictor of cognitive impairment.*

| **Biomarker** | **Ethnoracial coverage** | **Analytic N** | **Impaired n** | **OR (95% CI)** | **p** | **FDR-adjusted p** |
| --- | --- | --- | --- | --- | --- | --- |
| Plasma IL-6 | All three groups | 2,173 | 477 | 1.87 [1.31, 2.68] | <.001 | — |
| Plasma IL-5 | All three groups | 2,118 | 462 | 0.64 [0.46, 0.89] | .007 | .043 |
| Plasma IL-10 | All three groups | 2,166 | 471 | 0.99 [0.61, 1.58] | .950 | .950 |
| Plasma TNF-α | All three groups | 2,098 | 454 | 1.69 [0.87, 3.28] | .120 | .239 |

**Note.** Separate binary logistic regression models tested associations between each log10-transformed cytokine and cognitive impairment. Models adjusted for age, education, sex, ethnoracial group, Geriatric Depression Scale total score, and APOE ε4 carrier status. The prespecified primary biomarker was Simoa plasma IL-6; therefore, the FDR-adjusted p-value is intentionally blank for that row. Benjamini-Hochberg false discovery rate correction was applied across the six secondary cytokine models. ORs reflect a 10-fold increase in cytokine concentration. OR = odds ratio; CI = confidence interval; FDR = false discovery rate.

**Supplemental Table S9. Matched-sample sensitivity analyses examining IL-6, depressive symptoms, and cognitive impairment**

**Panel A. Black/White propensity score-matched sample (N = 554)**

| **Predictor** | **OR** | **95% CI** | **p** |
| --- | --- | --- | --- |
| Log10 IL-6 | 2.62 | 1.27–5.46 | .009 |
| GDS total score | 1.04 | 1.01–1.08 | .019 |
| Log10 IL-6 × ethnoracial group | 1.86 | 0.43–8.18 | .404 |
| GDS × ethnoracial group | 0.98 | 0.92–1.05 | .598 |

**Panel B. Hispanic/White propensity score-matched sample (N = 788)**

| **Predictor** | **OR** | **95% CI** | **p** |
| --- | --- | --- | --- |
| Log10 IL-6 | 2.24 | 1.27–3.97 | .005 |
| GDS total score | 1.06 | 1.03–1.10 | <.001 |
| Log10 IL-6 × ethnoracial group | 0.91 | 0.30–2.79 | .859 |
| GDS × ethnoracial group | 0.96 | 0.90–1.02 | .168 |

Note. Separate multivariable logistic regression models examined main effects and ethnoracial-group interaction terms within each matched sample. Models adjusted for age, education, sex, ethnoracial group, Geriatric Depression Scale (GDS) total score, and APOE ε4 carrier status. Cognitive impairment was defined as mild cognitive impairment or dementia versus cognitively unimpaired status. IL-6 was log10-transformed; odds ratios for IL-6 reflect a 10-fold increase in plasma IL-6 concentration. OR = odds ratio; CI = confidence interval.

**Supplemental Table S10. Sensitivity analyses examining chronic stress, plasma IL-6, and cognitive impairment**

**Panel A. Chronic stress as a predictor of log10-transformed plasma IL-6 (N = 2,168)**

| **Predictor** | **Beta** | **95% CI** | **p** |
| --- | --- | --- | --- |
| Chronic stress (per 5-point increase) | 0.021 | 0.011–0.031 | <.001 |

**Panel B. Cognitive impairment models before and after adjustment for chronic stress (N = 2,168)**

| **Model / predictor** | **OR** | **95% CI** | **p** |
| --- | --- | --- | --- |
| Model without chronic stress: log10 IL-6 | 1.76 | 1.24–2.51 | .002 |
| Model with chronic stress: log10 IL-6 | 1.79 | 1.26–2.56 | .001 |
| Model with chronic stress: chronic stress (per 5-point increase) | 0.96 | 0.88–1.04 | .330 |

**Note.** Panel A presents a linear regression model adjusted for age, education, sex, ethnoracial group, and Geriatric Depression Scale (GDS) total score. Panel B presents multivariable logistic regression models predicting cognitive impairment, defined as mild cognitive impairment or dementia versus cognitively unimpaired status. The models adjusted for age, education, sex, ethnoracial group, GDS total score, and plasma IL-6; the second model additionally included chronic stress. APOE ε4 carrier status was not available in the merged chronic-stress analytic dataset. OR = odds ratio; CI = confidence interval.
